# Opposing Role of Trust as a Modifier of COVID-19 Vaccine Uptake in an Indigenous Population

**DOI:** 10.3390/vaccines10060968

**Published:** 2022-06-17

**Authors:** Ruben Juarez, Krit Phankitnirundorn, May Okihiro, Alika K. Maunakea

**Affiliations:** 1 Department of Economics and UHERO, University of Hawaii, Honolulu, HI 96822, USA; kritphan@hawaii.edu; 2 Department of Anatomy, Biochemistry and Physiology, John A. Burns School of Medicine, University of Hawaii, Honolulu, HI 96813, USA; amaunake@hawaii.edu; 3 Department of Pediatrics, John A. Burns School of Medicine, University of Hawaii, Honolulu, HI 96813, USA; okihirom@hawaii.edu

**Keywords:** COVID-19, health disparities, trust, sources of information

## Abstract

Native Hawaiians and other Pacific Islanders (NHPIs) were disproportionately impacted by COVID-19 and remain significantly under-vaccinated against SARS-CoV-2. To understand vaccine hesitancy, we surveyed 1124 adults residing in a region with one of the lowest vaccination rates in Hawaii during our COVID-19 testing program. Probit regression analysis revealed that race/ethnicity was not directly associated with the probability of vaccine uptake. Instead, a higher degree of trust in official sources of COVID-19 information increased the probability of vaccination by 20.68%, whereas a higher trust in unofficial sources decreased the probability of vaccination by 12.49% per unit of trust. These results revealed a dual and opposing role of trust on vaccine uptake. Interestingly, NHPIs were the only racial/ethnic group to exhibit a significant positive association between trust in and consumption of unofficial sources of COVID-19 information, which explained the vaccine hesitancy observed in this indigenous population. These results offer novel insight relevant to COVID-19 mitigation efforts in minority populations.

## Structured Abstract

*Importance*: Native Hawaiians and other Pacific Islanders (NHPIs) were disproportionately impacted by COVID-19 and remain significantly under-vaccinated against SARS-CoV-2. Understanding vaccine hesitancy may improve vaccination uptake among NHPI and public health policy.
*Objective*: To examine how trust and COVID-19 information influence vaccine uptake in an indigenous population with low vaccination coverage disproportionately impacted by COVID-19.
*Design*: The NIH RADx-UP survey includes demographics, vaccination status, media consumption, and trust in sources of COVID-19 information and was collected from March to August 2021.
*Setting*: Adult residents of Hawaii completed an online survey via Qualtrics and participated in COVID-19 testing.
*Participants*: A total of 1124 adults participated in the RADx-UP survey: 61.7% self-identified as Native Hawaiian and Pacific Islander, 17.4% Asian, 13.0% Caucasian, and 7.7% Other; 63% were women; 29.0% were 18–29, 23.0% were 30–39, 21.0% were 40–49, 17.0% were 50–59, 7.3% were 60–69, and 2.8% were 70 years old or older.
*Main outcomes or measures*: Consumption of COVID-19 information, degree of trust in sources of COVID-19 information, and sociodemographic factors were measured in association with COVID-19 vaccine uptake.
*Results*: Among individuals that exhibited a higher degree of trust in official sources of COVID-19 information, we observed an increased probability of vaccination by 20.68%, whereas those that exhibited a higher degree of trust in unofficial sources had a decreased probability of vaccination by 12.49%, revealing a dual and opposing role of trust in vaccine uptake. Unlike age, sex, and education level, race/ethnicity was not an independent modifier of vaccine uptake. Trust in unofficial sources along with consumption of COVID-19 information from such sources explained vaccine hesitancy, specifically among NHPIs.
*Conclusion and Relevance*: The results offer novel insights into how the degree of trust in sources of COVID-19 information, frequency of consumption of COVID-19 information, and sociodemographic factors interact to influence vaccine uptake and offer novel insight relevant to public health policy in other similarly vulnerable minority populations in the US.

## 1. Introduction

The stagnating vaccination rates against SARS-CoV-2 and increased vulnerabilities to the more transmissible *Delta, Omicron,* and other emerging variants indicate the persistence of COVID-19 throughout the United States of America, especially among vaccine-hesitant populations [1]. Native Hawaiians and other Pacific Islanders (NHPIs) comprise 25% of Hawaii’s population, yet currently account for 38% of all COVID-19 cases [2], intensifying their pre-pandemic health disparities [3,4]. Indeed, long-standing social inequities have led to the disproportionately higher prevalence of and mortality to chronic diseases including obesity, diabetes, and heart disease among NHPIs compared to the overall population of the state [5,6,7,8]. Such pre-existing conditions render NHPIs particularly vulnerable to increased rates of SARS-CoV-2 infection and severe COVID-19 disease. Glaring gaps in vaccine coverage, historically derived sentiments of distrust in government, and the emergence of more infectious SARS-CoV-2 variants together fuel widening disparities even within the NHPI population [9].

The latest *Delta*- and *Omicron*-driven surges in cases threatened Hawaii’s healthcare system, with the majority (~90%) of COVID-19 hospitalizations arising among unvaccinated individuals and disproportionately impacting the NHPI population who remain under-vaccinated [6]. During this study, the vaccination rate among adult NHPIs was significantly lower at 51% than that of Whites (71%) and Asians (74%). Therefore, implementing public health policies to accelerate vaccine uptake and increase coverage among NHPIs is urgently needed.

To inform such policies, identifying contributors to vaccine hesitancy is a requisite. Although prior studies of other populations demonstrated that vaccine hesitancy is associated with distrust and misinformation [10,11], none have yet reported whether these factors account for the low vaccination rates in indigenous populations, including NHPIs. Additionally, the mechanism by which such factors might influence COVID-19 vaccine uptake remains unclear. Herein, we study how racial/ethnic differences in trust between sources of COVID-19 information coupled with the level of consumption of such information correspond to vaccine uptake. We anticipate that despite our focus on NHPIs, the mechanistic approach of our paper will offer novel insight into how these factors interact to influence vaccine uptake that will be generally applicable to public health policy in other similarly vulnerable minority populations in the US.

## 2. Methods

### 2.1. Recruitment Strategy

The study took place in a community in Hawaii with the highest density of Native Hawaiians per capita and has been implemented through a unique partnership between the University of Hawaii and five federally qualified health centers in the state called the Pacific Alliance Against COVID-19 (PAAC). PAAC is part of the National Institutes of Health (NIH) Rapid Acceleration of Diagnostics in Underserved Populations (RADx-UP) Initiative. All adult participants, 18 years old and older, were required to complete the RADx-UP survey either online via Qualtrics or a paper version, which was available at the testing site before getting a COVID-19 test. This survey included demographic data (e.g., self-reported age, sex, race/ethnicity) and vaccination status. The survey also included self-reported trust of various sources of information about COVID-19 (herein referred to as *trust*), the use or reliance on a source for information about COVID-19 (herein referred to as consumption), and other health-related behaviors, with a 66% completion rate of these metrics. These metrics have been vetted and used by the NIH RADx-UP initiative via a consortium of over 100 participating sites throughout the country [12]. Survey data were collected from March to August 2021, after COVID-19 vaccines were widely available for adults and before state mandates for vaccination were issued [13]. As summarized in Table 1 and Table 2, the proportion of racial/ethnic groups among the total of 1124 adult respondents of this survey was representative of the community population based on US 2020 Census data as well as their vaccination status at the time of the study (Zipcode: 96792). This study was approved by the Wai‘anae Coast Comprehensive Health Center Institutional Review Board.

### 2.2. Survey Instrument

Using the standardized NIH RADx-UP common data element, the degree of *trust* and frequency of *consumption* were obtained for a variety of information sources, including the federal and local government, healthcare providers, TV, radio, print or online news, friends, family members and other acquaintances, and contacts on social media. Trust-related questions were presented as: “How much do you trust [*source of information*] to provide correct information about COVID-19”? They were semi-quantified from the Likert scales 0–3: 0 represents “not trust at all”, 1 represents “a little”, 2 represents “somewhat”, 3 represents “a great deal”. Table 1 and Table 2 report the average values for each of the information sources measured broken down by vaccination status and by race/ethnicity.

Similarly, information consumption questions were presented as “How often do you use or rely on [*source of information*] to get information about the COVID-19 outbreak?” The values were semi-quantified as Likert scales 0–4: 0 represents “Never”, 1 represents “Rarely”, 2 represents “Sometimes”, 3 represents “Often”, and 4 “Always”. Table 1 and Table 2 report the average values for each of the information sources measured.

The official and unofficial trust and consumption indexes were computed as the average of trust and consumption within a subset of official and unofficial sources. Official sources included government, healthcare providers, and traditional channels of communication such as TV, radio, and print news; unofficial sources included social media channels, friends, family, acquaintances, and faith leaders. The average value of each of these indexes is shown in Table 1 and Table 2.

### 2.3. Statistical Analysis of Survey Data and Probabilistic Model

Ordinary least squares (OLS) estimations of average trust in official (or unofficial) sources on the frequency of consumption of information from official (or unofficial) sources, sex, racial/ethnic group, and education were performed using the following equation:

$$Trust=\beta_{1}+\alpha_{1}InformationConsumption+\beta_{2}Age+\beta_{3}Male+\beta_{4}Race+\;\beta_{5}Education+\mu$$

where Trust refers to average trust in official (or unofficial) index, InformationConsumption refers to the frequency of consumption of information from official (or unofficial), Age refers to the age (in years old) of a participant, Male is a dummy variable indicating whether the participant is male (Male = 1) or not (Male = 0), Race is three dummy variables referring to the participant’s racial/ethnic group (NHPI, Asia, or Others) with White as the baseline, and Education is four dummy variables referring to the participant’s level of education (High school graduate or GED, Some college level, Bachelor’s degree, and Other advance degrees) with ‘Not completed high school.’ as a baseline. Estimations of the coefficients are presented in Table 3 columns a and c for the indicated demographic variables. Table 3b represents a Probit regression [14] for estimating vaccination status as a function of trust in official and unofficial sources using the following equation:

$$Vaccination=\beta_{1}+\alpha_{1}TrustOfficial+\alpha_{2}TrustUnofficial+\beta_{2}Age+\;\beta_{3}Male+\beta_{4}Race+\beta_{5}Education+\mu$$

where Vaccination refers to the vaccination status of the participant. If the participant was vaccinated, Vaccination is equal to 1 and Vaccination is equal to 0 if the participant was not vaccinated. TrustOfficial refers to the average trust in official sources and TrustUnofficial refers to the average trust in unofficial sources. Age, Male, Race, and Education represent the same variables mentioned above. The accuracy of the Probit regression was measured as the percentage of correctly predicted vaccination outcomes, resulting in 70.5% correctly predicted outcomes (31.8% of overall outcomes when adjusted for the most frequent outcomes). These analyses were completed using R Statistical Software version 4.0.5 (R Project for Statistical Computing) within RStudio statistical software version 1.3.1073 (RStudio).

## 3. Results

### 3.1. Opposing Role of Trust in Modifying Vaccine Hesitancy

To determine the extent to which trust influenced vaccine uptake, we first examined perceptions of trust in official or unofficial sources of COVID-19 information as well as the frequency of consumption of COVID-19 information from official or unofficial sources. Data was stratified based on vaccination status, age, sex, racial/ethnic group, and level of education. We observed that compared to unvaccinated individuals, those that were vaccinated reported a higher degree of trust in official sources of COVID-19 information, concomitant with more frequent consumption of this information (Table 1). These results implicated trust and consumption of COVID-19 information as co-dependent modifiers of vaccine uptake where the choice regarding vaccination varied depending on the frequency of consumption of COVID-19 information from official or unofficial sources. Indeed, we observed a significant positive linear relationship between the frequency of consumption of COVID-19 information from either official or unofficial sources and the degree of trust in those sources, respectively (0.671, *R*^2^ = 0.44, *p* < 0.001, 95% CI = (0.46, 0.699) and 0.375, *R*^2^ = 0.19, *p* < 0.001, 95% CI = (0.089, 0.284); Table 3a,c).

By comparing vaccination status and trust, we found a significant positive association between uptake of the COVID-19 vaccine and degree of trust in official sources (0.532, *p* < 0.001, 95% CI = (0.415, 0.651); Table 3b). In contrast, we observed a significant negative association between vaccine uptake and degree of trust in unofficial sources (−0.321, *p* < 0.001, 95% CI = (−0.449, −0.195); Table 3b). These results indicate that individuals with a higher degree of trust in official sources of COVID-19 information were more receptive to receiving the COVID-19 vaccine, whereas those with a higher degree of trust in unofficial sources were less receptive, revealing an opposing role of trust in vaccine uptake. To highlight this opposing role, we estimated the probability of vaccine uptake using the marginal effect of the Probit regression model [15]. We computed that, on average, increasing one unit of trust (as on the Likert scale) in *official* sources increased the probability of vaccination by 20.68%, whereas an increase of one unit of trust in *unofficial* sources decreased the probability of vaccination by 12.49%. This result indicated that perceived trust indeed plays an opposing role in modifying vaccine uptake, the outcome and degree to which was dependent on the source of COVID-19 information.

### 3.2. Race/Ethnicity Alone Is Not a Modifier of Vaccine Uptake in NHPIs

We next examined other potential modifiers of vaccine hesitancy. Given the significant racial/ethnic differences in vaccine uptake, especially among Native Hawaiians in Hawaii who remained under-vaccinated [16], we expected to observe race/ethnicity as a modifier. Surprisingly, with the only exception among the Other racial/ethnic group, we observed that race/ethnicity alone was not directly associated with the probability of vaccine uptake (−0.013, *p* = 0.92, 95% CI = (−0.284, 0.029), NHPI; and 0.223, *p* = 0.16, 95% CI = (−0.758, 0.538), Asians; Table 3b). Moreover, among NHPIs, our data indicated that trust in official and unofficial sources along with race/ethnicity modify vaccine uptake. Compared to Whites, NHPIs reported a higher degree of trust in unofficial sources by 1.21 (21% higher than Whites) and more frequent consumption of unofficial COVID-19-related information by 1.28 (22% higher than Whites) (*t*-test, *p* = 0.007, 95% CI = (−0.363, −0.0591); and *t*-test, *p* = 0.01, 95% CI = (−0.407, −0.0562); Table 2). Interestingly, we observed a significant positive association between the degree of trust in and frequency of consumption of COVID-19 information from unofficial sources only among NHPIs relative to Whites (0.177, *p* = 0.029, 95% CI = (0.0414,0.294); Table 3c). While the lack of trust in official government sources of information observed was anticipated based on historical and present-day political, social, and structural discrimination of NHPIs [4,17,18], the significantly higher degree of trust in unofficial sources of COVID-19 information was not expected.

### 3.3. Age, Sex, and Education Level as Co-Modifiers of Vaccine Uptake

Finally, we found that overall, age had a strong effect on vaccination uptake and a weak effect on the association between the degree of trust and consumption of COVID-19 information by unofficial sources (0.023, *p* < 0.01, 95% CI = (0.017, 0.029); 0.004, *p* < 0.03, 95% CI = (0.0002, 0.00797); Table 3b,c) and that males had a higher degree of trust in official, but not unofficial, sources of information relative to females (0.144, *p* < 0.01, 95% CI = (0.022205, 0.235); and −0.028, *p* = 0.51, 95% CI = (−0.139, 0.084); Table 3a,c). In addition, we observed that level of education was not a contributor to the degree of trust in official or unofficial sources (Table 3a,c).

However, the level of education was significantly associated with vaccine uptake independent of other factors relative to individuals with no high school diploma (0.571, *p* = 0.06, 95% CI = (0.165, 0.986); 0.962, *p* < 0.001 95% CI = (0.31, 1.40); and 1.132, *p* < 0.001, 95% CI = (0.710, 1.57); Table 3b) for college level, Bachelor’s, and other advanced degrees, respectively, indicating that education may act as a modifier of vaccine hesitancy. Indeed, when accounting for education level, we no longer observed a significant association between the degree of trust and frequency of consumption of COVID-19 information from official sources among NHPIs (−0.127, *p* = 0.30, 95% CI = (−0.281, 0.027); Table 3a). These results, coupled with the negative association between the degree of trust in unofficial sources and vaccine uptake (−0.013, *p* = 0.03, 95% CI = (−0.449, −0.195); Table 3c), indicate that race/ethnicity is notable but insufficient alone as a modifier of vaccine hesitancy among NHPIs and instead must be considered along with education, trust, and consumption of COVID-19 information. Therefore, accounting for these covariates, the lower vaccine uptake among NHPIs appears to be primarily driven by their higher degree of trust in and consumption of COVID-19 information from unofficial sources.

## 4. Discussion

Vaccine hesitancy is a significant barrier to decreasing the spread and severity of SARS-CoV-2 viral infections and ending the COVID-19 pandemic. Thus, a better understanding of the underlying contributors to vaccine hesitancy, especially of social factors that influence individual-level choice and decision making that ultimately lead to vaccine uptake outcomes, is of critical public health importance. Altogether, our data offers a new model for how trust in and consumption of COVID-19 information from distinct sources modifies vaccine uptake at the individual level, highlighting novel opportunities for addressing vaccine hesitancy at the population level. Importantly, we show that trust has an opposing role in modifying vaccine uptake, which is highly dependent on the source of information. Our finding that trust in official sources of information, the degree of which is correlated with the frequency of consumption of such information, is a positive modifier of vaccine uptake highlights the importance of effective communication strategies by the government and other key sources of information. This is consistent with previous research that demonstrated how public trust in governments across the world is associated with vaccine uptake [19,20]. Our findings are also consistent with the literature that has demonstrated that health care and doctors, as well as other medical sources such as the CDC, are trusted sources of information about the pandemic and the COVID-19 vaccine [21,22,23,24,25].

Our findings also indicate that trust in unofficial sources of information, the degree of which is correlated with the frequency of consumption of such information, is a negative modifier of vaccine uptake. Pertwee et al. [26], who studied vaccine hesitation across many cultures and countries, expressed that beliefs in conspiracy theories and false information about COVID-19 and vaccines should be “read as expressions of popular fears and anxieties”. Conspiracy theories, including the various narratives that promote vaccine misinformation, arise and spread during times of uncertainty, which the long pandemic has cultivated. These false narratives often vastly simplify complex issues, such as the risk vs. benefits of emerging COVID-19 vaccinations, widening their appeal to lay people, especially those prone to distrust in government (official sources). Thus, the negative relationship we found between trust in unofficial sources and vaccine uptake may indicate that such sources are actively discouraging vaccination by spreading misinformation. These sources likely also promote vaccine hesitation indirectly by stoking doubt in official sources of information [11,27].

In contrast with the above literature that focuses on specific metrics of trust or consumption in official or unofficial sources, our results indicate a novel approach that quantifies the likelihood of vaccine uptake using a new composite metric of official and unofficial consumption and trust in information sources. We anticipate that these composite metrics may be more accurate than specific trust and/or consumption metrics at estimating vaccine uptake.

Vaccine hesitancy persists across various communities throughout the world [28] and has been widely documented in Canada [29,30], India [31,32], Sudan [33], and Yemen [34]. Disparities in vaccine uptake in the US, especially among minorities, have also been well documented, including among Hispanics and Blacks [35], and during the course of this study, indigenous populations including Native Hawaiian and other Pacific Islanders [9,36,37,38]. Given the significant under-vaccination among NHPIs in Hawaii at the time of our study, we expected to observe race/ethnicity as a direct modifier of vaccine uptake. Instead, our results showed that trust in official and unofficial sources along with race/ethnicity act as a mediator of vaccine uptake. Interestingly, we observed a significant positive association between the degree of trust in and frequency of consumption of COVID-19 information from unofficial sources only among NHPIs relative to Whites. While trust in official, government sources of information observed was anticipated based on historical and present-day political, social, and structural discrimination of NHPIs [4,17,18], the significantly higher degree of trust in unofficial sources of COVID-19 information was not expected. These results indicate that it is not racial/ethnic differences themselves that are associated with vaccine hesitancy, but that they must be contextualized with individual-level degrees of trust in and frequency of COVID-19 information consumption. These results have significant implications on how to interpret results of the variability in vaccine hesitancy to consider additional factors beyond race/ethnicity.

Finally, our results show that age had a weak effect on the association between the degree of trust and consumption of COVID-19 information for both official and unofficial sources. This is consistent with the previous literature reporting that younger individuals tend to be under-vaccinated relative to older individuals [39,40]. Our result is also consistent with previous studies that show slight differences in vaccine uptake among males and females [40]. In contrast with these studies, we show that these sex differences are due to trust; in particular, we show that males had a higher degree of trust and consumption of official, but not unofficial, sources of information relative to females. Although differences in vaccine uptake across education levels had been well documented in the literature [36,41,42], our study is the first to show that level of education was not a contributor to the degree of trust in official or unofficial sources. Collectively, accounting for these covariates, the lower vaccine uptake among NHPIs appears to be primarily driven by their higher degree of trust in and consumption of COVID-19 information from unofficial sources.

### 4.1. Limitations

As our recruitment strategy was restricted to one community in Hawaii, the predominant racial/ethnic group in our dataset was Native Hawaiians (58%). Although generally, the proportion of major racial/ethnic groups such as Asians and Whites closely resembles the composition of the community, our dataset comprised a small number of individuals from non-Native Hawaiian minority racial/ethnic groups. Indeed, only 3% of Pacific Islanders categorized within NHPIs were in our dataset, and Other racial/ethnic groups that are considered minorities in Hawaii (e.g., Hispanics, Blacks, and Native Americans) collectively made up just 7%, aligning with the composition in the state of Hawaii. Given this limitation, we continued to apply the 1997 US Office of Management and Budget reclassification of Native Hawaiians under the NHPI category and combined racial/ethnic minorities under Other.

We recognize the importance of disaggregating data to measure racial/ethnic disparities in COVID-19 [16,43]. Indeed, disaggregated data of vaccination rates among NHPIs revealed that while PIs were covered at the same level representative of their population (4%), NHs were under-vaccinated at 14% (versus 21% of the population) [3]. Coupled with the higher transmissibility of the *Delta* variant, this low vaccination coverage contributed to the over-representation of NHs in infections, accounting for 29% of all COVID-19 cases at the time of this writing, while the representative vaccination coverage among PIs was associated with a significant decline in COVID-19 among this population [2]. Therefore, given that our dataset largely comprised NHs, we are confident that the aggregated data of NHPIs well represents the NH population and is relevant to COVID-19 vaccine hesitancy. However, any variability of the metrics collected among PIs will be masked by that of NHs, and the low sample size of other racial/ethnic minority groups represented precluded a powered disaggregated analysis.

Another limitation of this study is that our model does not account for other factors such as income, household size, job type/sector, risk of COVID-19 exposure, pre-existing medical conditions, etc., that may influence vaccine uptake. Indeed, a recent study of a limited number of patients with neurological disorders in Hawaii examined over 30 sociodemographic variables and medical comorbidities and found that among NHPI patients, a positive depression screening reduced the odds of vaccine acceptance [44]. Thus, we determined the accuracy of our model that considered age, sex, education, and trust and consumption of COVID-19 information by calculating the ratio of correctly predicted outcomes in vaccine uptake over our total number of observations (n = 1124). We found that our Probit model correctly predicted 70.5% of overall outcomes with just these five factors alone. However, additional studies to examine the degree to which other potential modifiers might improve the accuracy of our model are warranted.

### 4.2. Conclusions and Future Directions

The recent, amplified resurgence of COVID-19 in Hawaii underscores the adverse consequences of long-standing yet unaddressed social inequities in its indigenous population, highlighting the importance and wide-reaching benefits of establishing health equity. The results of our study offer insight into the nuances of vaccine hesitancy with which community and culturally relevant interventions may be tailored to increase trust in key community organizations and reduce disparities related to COVID-19.

Further investigation to confirm the specific constituents that comprise unofficial sources of information is therefore expected to reveal additional insight into vaccine hesitancy. As new COVID-19 surges emerge and threaten other states [45] and countries, addressing vaccine hesitancy becomes an ever more pressing priority for COVID-19 mitigation policies. Our data suggest that interventions that foster trust in official sources of COVID-19 information and promote health literacy may more effectively increase vaccine uptake.

Despite our focus on NHPIs, the social factors and the mechanism uncovered in our analyses offer novel insight into how these factors interact to influence vaccine uptake. We anticipate these results will be useful in shaping and tailoring public health policy for other similarly vulnerable minority populations in the US.

## Figures and Tables

**Table 1:** Summary statistics of responses to RADx-UP survey broken down by vaccination status.

Demographics	Overall, N = 1124		Vaccinated, N = 637		Unvaccinated, N = 487	
**Vaccination status**	**N**	**%**	**N**	**%**	**N**	**%**
Vaccinated	637	57.0%	NA	NA	NA	NA
**Age Group**	**N**	**%**	**N**	**%**	**N**	**%**
18–29	323	29.0%	136	21.0%	187	38.0%
30–39	254	23.0%	129	20.0%	125	26.0%
40–49	240	21.0%	143	22.0%	97	20.0%
50–59	193	17.0%	137	22.0%	56	11.0%
60–69	82	7.3%	62	9.7%	20	4.1%
70+	32	2.8%	30	4.7%	2	0.4%
**Sex**	**N**	**%**	**Mean**	**%**	**N**	**%**
Female	708	63.0%	408	64.0%	300	61.0%
Male	416	37.0%	229	36.0%	187	38.0%
**Education**	**N**	**%**	**N**	**%**	**N**	**%**
9th to 12th grade, no diploma	54	4.8%	18	2.8%	36	7.4%
High school graduate or GED completed	407	36.0%	164	26.0%	243	50.0%
Some college level/Technical/Vocational degree	269	24.0%	159	25.0%	110	23.0%
Bachelor’s degree	174	15.0%	121	19.0%	53	11.0%
Other advanced degree (Master’s, Doctoral degree)	220	20.0%	175	27.0%	45	9.2%
**Trust—Official Source of COVID-19 Information ^1^**	**Mean**	**SD**	**Mean**	**SD**	**Mean**	**SD**
U.S. Government	1.18	1.10	1.42	1.12	0.85	0.98
U.S. Coronavirus task force	1.32	1.17	1.55	1.21	1.01	1.04
Doctor or health care provider	1.92	1.23	2.07	1.24	1.74	1.18
News on the radio, TV, online, or in newspapers	1.17	1.05	1.36	1.06	0.92	0.99
**Average overall**	**1.40**	**1.00**	1.60 ***	**1.05**	**1.13**	**0.87**
**Trust—Unofficial Source of COVID-19 Information ^1^**	**Mean**	**SD**	**Mean**	**SD**	**Mean**	**SD**
Faith leaders	1.09	1.24	1.00	1.23	1.21	1.25
Close friends and family members	1.56	1.15	1.58	1.15	1.53	1.16
Classmates, colleagues or other people you know	1.27	1.06	1.34	1.07	1.19	1.04
Contacts on social media	0.80	0.94	0.82	0.96	0.76	0.91
**Average overall**	**1.18**	**0.89**	**1.19**	**0.90**	**1.17**	**0.86**
**Consumption—Official Source of COVID-19 Information ^2^**						
Local government officials (e.g., Governor, Mayor)	1.47	1.17	1.66	1.21	1.23	1.07
Federal government (e.g., President, White House Coronavirus Task Force)	1.44	1.17	1.64	1.22	1.19	1.05
Print or online news	1.40	1.13	1.52	1.17	1.24	1.04
TV or radio	1.36	1.12	1.48	1.16	1.20	1.04
Healthcare providers (e.g., Personal Physician/Doctor, Pharmacist, etc.)	1.79	1.27	1.88	1.34	1.68	1.17
Medical/Health websites (e.g., CDC, WebMD, etc.)	1.65	1.21	1.77	1.26	1.48	1.11
**Average overall**	**1.52**	**1.01**	1.66 **	**1.07**	**1.34**	**0.91**
**Consumption—Unofficial Source of COVID-19 Information ^2^**						
Friends, family or neighbors (not including social media)	1.43	1.14	1.38	1.15	1.50	1.13
Social media (e.g., Instagram, Facebook, YouTube, TikTok, etc.)	1.05	1.08	1.03	1.10	1.08	1.05
**Average overall**	**1.24**	**0.98**	**1.21**	**1.01**	**1.29**	**0.95**

^1^ Question: How much do you trust each of these sources to provide correct information about COVID-19? Answers provided on a liker scale:—Not at all (0), A little (1), Somewhat (2) and A great deal (3); Reported values represent the mean of the liker scale while the standard deviation is shown inside the parenthesis. ^2^ Question: How often do you use or rely on the following sources to get information about the COVID-19 outbreak? Answers provided on a liker scale—Never (0), Rarely (1), Sometimes (2), Often (3) and Always (4). Reported values represent the mean of the liker scale while the standard deviation is shown inside the parenthesis. **, *** indicates pairwise statistical significance at *p* < 0.05, *p* < 0.01 by two-tailed t-test between the vaccinated vs unvaccinated groups.

**Table 2:** Summary statistics of responses to RADx-UP survey broken down by race/ethnicity.

Demographics	NHPI, N = 694		Asian, N = 196		White, N = 147		Other, N = 87	
**Vaccination status**	**N**	**%**	**N**	**%**	**N**	**%**	**N**	**%**
Vaccinated	352	51.0%	146	74.0%	104	71.0%	35	40.0%
**Age Group**	**N**	**%**	**N**	**%**	**N**	**%**	**N**	**%**
18–29	222	32.0%	43	22.0%	31	21.0%	27	31.0%
30–39	164	24.0%	41	21.0%	29	20.0%	20	23.0%
40–49	150	22.0%	39	20.0%	33	22.0%	18	21.0%
50–59	107	15.0%	43	22.0%	29	20.0%	14	16.0%
60–69	34	4.9%	25	13.0%	16	11.0%	7	8.0%
70+	17	2.4%	5	2.6%	9	6.1%	1	1.1%
**Gender**	**N**	**%**	**N**	**%**	**N**	**%**	**N**	**%**
Female	451	65.0%	124	63.0%	93	63.0%	40	46.0%
Male	243	35.0%	72	36.0%	54	37.0%	47	54.0%
**Education**	**N**	**%**	**N**	**%**	**N**	**%**	**N**	**%**
9th to 12th grade, no diploma	42	6.1%	1	0.5%	6	4.1%	5	5.7%
High school graduate or GED completed	322	46.0%	37	19.0%	16	11.0%	32	37.0%
Some college level/Technical/Vocational degree	169	24.0%	53	27.0%	26	18.0%	21	24.0%
Bachelor’s degree	77	11.0%	46	23.0%	36	24.0%	15	17.0%
Other advanced degree (Master’s, Doctoral degree)	84	12.0%	59	30.0%	63	43.0%	14	16.0%
**Trust—Official Source of COVID-19 Information ^1^**	**Mean**	**SD**	**Mean**	**SD**	**Mean**	**SD**	**Mean**	**SD**
U.S. Government	1.11	1.06	1.39	1.14	1.30	1.2	1.02	1.06
U.S. Coronavirus task force	1.26	1.13	1.47	1.20	1.46	1.29	1.09	1.14
Doctor or health care provider	1.89	1.22	2.04	1.21	1.92	1.28	1.89	1.21
News on the radio, TV, online, or in newspapers	1.17	1.05	1.28	1.05	1.14	1.09	0.99	1.03
**Average overall**	**1.36**	**0.98**	**1.55**	**1.04**	**1.46**	**1.09**	**1.25**	**0.94**
**Trust—Unofficial Source of COVID-19 Information ^1^**	**Mean**	**SD**	**Mean**	**SD**	**Mean**	**SD**	**Mean**	**SD**
Faith leaders	1.18	1.28	0.98	1.17	0.65	1.04	1.36	1.24
Close friends and family members	1.58	1.17	1.59	1.12	1.37	1.10	1.63	1.17
Classmates, colleagues or other people you know	1.27	1.08	1.34	1.06	1.20	1.01	1.26	1.03
Contacts on social media	0.81	0.94	0.77	0.94	0.77	0.94	0.75	0.93
**Average overall**	**1.21 ^ttt^**	**0.90**	**1.17 ^t^**	**0.85**	**1.00**	**0.84**	**1.25 ^tt^**	**0.87**
**Consumption—Official Source of COVID-19 Information ^2^**								
Local government officials (e.g., Governor, Mayor)	1.44	1.17	1.63	1.19	1.52	1.21	1.36	1.08
Federal government (e.g., President, White House Coronavirus Task Force)	1.42	1.15	1.55	1.21	1.51	1.27	1.31	1.09
Print or online news	1.40	1.13	1.42	1.10	1.38	1.2	1.41	1.08
TV or radio	1.38	1.15	1.39	1.07	1.24	1.11	1.26	1.04
Healthcare providers (e.g., Personal Physician/Doctor, Pharmacist, etc.)	1.84	1.26	1.74	1.28	1.66	1.29	1.71	1.28
Medical/Health websites (e.g., CDC, WebMD, etc.)	1.64	1.20	1.69	1.20	1.65	1.31	1.60	1.18
**Average overall**	**1.52**	**1.02**	**1.57**	**1.01**	**1.49**	**1.06**	**1.44**	**0.92**
**Consumption—Unofficial Source of COVID-19 Information ^2^**								
Friends, family or neighbors (not including social media)	1.47	1.14	1.35	1.08	1.24	1.11	1.64	1.28
Social media (e.g., Instagram, Facebook, YouTube, TikTok, etc.)	1.08	1.07	0.97	1.03	0.86	1.09	1.29	1.16
**Average overall**	**1.28 ^ttt^**	**0.98**	**1.16**	**0.94**	**1.05**	**0.98**	**1.47 ^ttt^**	**1.09**

^1^ Question: How much do you trust each of these sources to provide correct information about COVID-19? Answers provided on a liker scale:—Not at all (0), A little (1), Somewhat (2) and A great deal (3); Reported values represent the mean of the liker scale while the standard deviation is shown inside the parenthesis. ^2^ Question: How often do you use or rely on the following sources to get information about the COVID-19 outbreak? Answers provided on a liker scale—Never (0), Rarely (1), Sometimes (2), Often (3) and Always (4). Reported values represent the mean of the liker scale while the standard deviation is shown inside the parenthesis. ^t^, ^tt^, ^ttt^ indicate pairwise statistical significance at *p* < 0.1, *p* < 0.05, *p* < 0.01 respectively by two-tailed *t*-test between each shown race/ethnic group vs the white race/ethnic group.

**Table 3:** The mechanism for vaccine uptake as a function of Trust and Consumption in Official and Unofficial sources. From left to right: (a) OLS Regression of Trust-Official Source as the dependent variable, Consumption-Official Source and demographics as independent variables; (b) Probit regression of Vaccination Status as the dependent variable, and Trust-Official Source, Trust Unofficial Source, and demographics as independent variables; (c) OLS regression of Trust-Unofficial Source as the dependent variable, Consumption-Unofficial Source and demographics as independent variables.

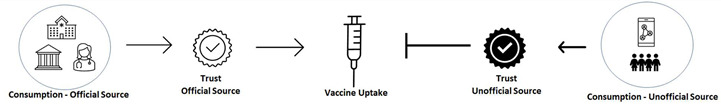					
(a)		(b)		(c)	
**Trust—Official Source of COVID-19 Information ^†^**		**Received COVID-19 Vaccine ^^^**		**Trust—Unofficial Source of COVID-19 Information ^‡^**	
**Consumption—Official Source**	0.671*** (0.030)	**Trust—Official Source ^1^**	0.532 *** (0.060)	**Consumption—Unofficial Source**	0.375 *** (0.029)
		**Trust—Unofficial Source ^1^**	−0.321 *** (0.065)		
Age	0.003 (0.002)	Age	0.023 *** (0.003)	Age	0.004 ** (0.002)
**Race/Ethnicity—NHPI**	−0.127 (0.079)	**Race/Ethnicity—NHPI**	−0.013 (0.138)	**Race/Ethnicity—NHPI**	0.177 ** (0.085)
Race/Ethnicity—Asian	0.052 (0.090)	Race/Ethnicity—Asian	0.223 (0.160)	Race/Ethnicity—Asian	0.170 * (0.097)
Race/Ethnicity—Other	−0.279 ** (0.118)	Race/Ethnicity—Other	−0.381 ** (0.192)	Race/Ethnicity—Other	0.068 (0.128)
Gender—Male	0.114 ** (0.052)	Gender—Male	0.030 (0.087)	Gender—Male	−0.028 (0.057)
Education—High school graduate or GED completed	−0.043 (0.127)	Education—High school graduate or GED completed	0.206 (0.202)	Education—High school graduate or GED completed	−0.033 (0.138)
Education—Some college level/Technical/Vocational degree	−0.010 (0.131)	Education—Some college level/Technical/Vocational degree	0.571 *** (0.209)	Education—Some college level/Technical/Vocational degree	0.076 (0.142)
Education—Bachelor’s degree	0.055 (0.138)	Education—Bachelor’s degree	0.962 *** (0.222)	Education—Bachelor’s degree	0.081 (0.150)
Education—Other advanced degree (Master’s, Doctoral degree)	0.115 (0.136)	Education—Other advanced degree (Master’s, Doctoral degree)	1.132 *** (0.222)	Education—Other advanced degree (Master’s, Doctoral degree)	0.044 (0.148)
Constant	0.417 ** (0.164)	Constant	−1.430 *** (0.272)	Constant	0.674 *** (0.178)
Observations	752	Observations	1.124	Observations	752
R^2^	0.444	Log Likelihood	−622.168	R^2^	0.193
Adjusted R^2^	0.437	Akaike Inf. Crit.	1.268.337	Adjusted R^2^	0.182
Residual Std. Error	0.668 (df = 741)			Residual Std. Error	0.724 (df = 730)
F Statistic	59.207 *** (df = 10; 741)			F Statistic	17.694 *** (df = 10; 741)

^†^ OLS Regressions with **Trust—Official Source** as a dependent variable and **Consumption—Official Source** as an independent variable as well as other demographic variables. ^‡^ OLS Regressions with **Trust—Unofficial Source** as a dependent variable and **Consumption—Unofficial Source** as an independent variable as well as other demographic variables. ^^^ Probit Regressions with **Vaccination Uptake** (1 vaccinated, 0 not vaccinated) as dependent variable, and **Trust—Official/Unofficial Sources** as independent variables as well as other demographic variables. From regression analyses indicated, significant ***p*****-values** are denoted by * *p* < 0.10; ** *p* < 0.05; *** *p* < 0.01. ^1^ The coefficient for Trust—Official Source (0.532) equals to an average 20.68% increase probability of vaccination per unit of trust using the marginal effect of Probit regression. Similarly, the coefficient for Trust—Unofficial Source (−0.321) equals to an average 12.49% decrease probability of vaccination per unit of trust using the marginal effect of Probit regression.

## Data Availability

All data used for this project will be available de-identified as approved by the Waianae Coast Comprehensive Health Center Institutional Review Board upon request to the corresponding author.
